# TVSEP: A comprehensive long-term panel dataset on shocks, welfare and agriculture in rural Thailand and Vietnam

**DOI:** 10.1016/j.dib.2026.112993

**Published:** 2026-06-19

**Authors:** Ulrike Grote, Trung Thanh Nguyen, Manh Hung Do

**Affiliations:** Institute for Environmental Economics and World Trade, Leibniz University Hannover, Königsworther Platz 1, 30167 Hannover, Germany

**Keywords:** Data quality, Poverty, Climate change, COVID-19, Shocks, Migration, Southeast Asia

## Abstract

The Thailand Vietnam Socio-Economic Panel (TVSEP) is a panel dataset which assists in analyzing long-term behaviors and welfare developments of households and individuals in rural regions that are vulnerable to diverse shocks such as droughts, floods, price fluctuations or health shocks. TVSEP covers 2200 rural households from 220 villages in Thailand and Vietnam, and to date, ten waves have been collected from 2007 to 2024. The paper explains the panel dataset and highlights some trends and scientific contributions in agricultural and development economics. Apart from different types of shocks and coping behavior of households, the data also includes information on the household members, on well-being and aspirations, personal satisfaction and character traits, income generated from land, crops, livestock and natural resources, on wage and non-farm self-employment, financial aspects, household expenditures and consumption, but also assets and housing conditions, access to information and communication technology (ICT). The migrant tracking survey allows to link remittances sent by household members to their rural households. In sum, TVSEP data not only provide an important empirical basis for analyzing long-term future trends and patterns, but also enable deeper insight into socioeconomic processes and behaviors, thereby providing policymakers with more informed guidance.

Specifications Table

**Table:** 

Subject	Social Sciences
Specific subject area	Socio-Economic Panel: Rural livelihoods, rural transformation and behaviors and developments of households and individuals, including migrants, facing different types of shocks, in Thailand and Vietnam
Type of data	Raw datasets are available in STATA data format (i.e., “.dta” format). Supporting materials: description of survey, questionnaires and guidelines for training and cleaning (available at https://www.tvsep.de/en/survey-documents).
Data collection	Household and village head surveys were conducted in three provinces of Thailand (Nakhon Phanom, Ubon Ratchathani, Buriram) and three provinces of Vietnam (Ha Tinh, Thua Thien Hue, Dak Lak). The migrant tracking surveys were conducted in the Greater Bangkok Area in Thailand and in Ho Chi Minh City (HCMC) and surroundings as well as Da Nang City and also in Hanoi in Vietnam. Data collection was conducted by partners from Universities in Thailand and Vietnam. They recruit enumerators who are intensively trained. The interviews were conducted face-to-face in the rural areas or close to the migrants’ residences or work places in the cities. Since 2016, the questionnaires are computed in survey solution, an open access software provided by the World Bank, and computer-assisted personal interviews (CAPI) are applied. Before, paper and pencil interviews (PAPI) were used. For the rural household surveys, the provinces were purposively selected. To identify subdistricts/communes, villages and eventually households, a 3-stage cluster sampling procedure was applied. Information about questionnaires used in each survey wave can be found at: https://www.tvsep.de/en/survey-documents#c80720 (e.g., for 2024).
Data source location	The raw datasets are stored on a secure server located at the Leibniz University Hannover (LUH) (encrypted using password protection), maintained by the Leibniz Universität IT Services (LUIS).
Data accessibility	Repository name: TVSEP Direct URL to data: https://www.tvsep.de/en/tvsep-data-access TVSEP data is primary, open access data, available free of charge for research and teaching purposes. The data is made available to the researcher through a Data Usage Agreement (DUA) with the Institute for Environmental Economics and World Trade, Leibniz University Hannover (LUH). The DUA requires the name and affiliation of the applicant and a working title of the envisaged research. The data is received upon signing the DUA provided on the TVSEP homepage. The DUA template is included in the supplemental materials and can be also directly accessed at https://www.tvsep.de/en/form. A sample of the TVSEP data which was extracted from the raw datasets can be downloaded without any restrictions at https://www.tvsep.de/en/data/sample-of-the-tvsep-dataset.
Related research article	By the end of 2025, around 150 peer-reviewed journal articles and 57 dissertations were published in the context of the TVSEP project. Direct URL to publications: https://www.tvsep.de/en/publications-1 or to research topics https://www.tvsep.de/en/research/research-topics

## 1 Value of the Data

• There is a lack of updated panel data for the Global South. TVSEP data is valuable as it has large sample sizes of 4400 households from Thailand and Vietnam which helps to produce more reliable results. The dataset is also very comprehensive containing information on shocks and risks to which in particular rural households are exposed, crop/plot-level data for crop production analyses, and diverse households’ livelihood strategies. It also covers topics such as rural-urban and urban-rural migration, agricultural transformation, or intergenerational aspects of households. The data from the migrant tracking survey allows to link remittances from the migrants with their rural households and vice versa (during the COVID-19 pandemic).
• The data covers a long time period (2007–2024) allowing to identify poverty and welfare trends, dynamics and patterns. Identifying strategies and trends leads to more general and robust results, enabling a broader, more global perspective. Over time, a core set of questions remained stable to ensure consistency over time. However, additional upcoming topics were added reflecting advances in research, new environmental challenges and technological developments. Furthermore, the attrition rates in the TVSEP project are quite low, which is impressive given that (i) the panel has been ongoing for a long time and (ii) the difficulties of collecting data from rural populations in developing countries.
• The panel allows for comparisons between the two emerging economies Thailand and Vietnam. While Thailand is an upper middle-income country, Vietnam achieved the lower middle-income status in 2011 but might advance to the upper middle-income group in 2026. The data can serve to test hypotheses concerning household behavior, livelihood strategies or structural changes across the two neighboring countries. Identifying strategies and trends leads to more general and robust results, enabling a broader, more global perspective.
• Rural areas are rapidly transforming, natural resources are significantly degrading and climate change is more severely and frequently impacting on the growing populations and economies of Southeast Asia, particularly in Thailand and Vietnam. These trends affect the livelihoods and farming activities of rural populations, which could ultimately jeopardize food security and lead to greater poverty in the future. It is therefore extremely important to conduct research which guides policymakers in designing policies to help people cope with shocks in Thailand and Vietnam. TVSEP data offers not only a crucial empirical basis for analyzing long-term trends and patterns, but also provides a deeper insight into socioeconomic processes and behaviors which help households to cope with shocks and safeguard their livelihoods.
• TVSEP data can be used for research and education to replicate existing studies and conduct further analyses. While a number of peer-reviewed publications have already used the TVSEP data, there is still room for advancing research by using different methodologies and on diverse topics such as migration and mobility, inequalities, behavioral aspects including attitudes and life satisfaction or life course development, which refers to the development of people’s lives within their socio-economic, cultural contexts. Given its importance for examining welfare dynamics of the rural population over time and for comparing welfare trends in two emerging economies, continuing the TVSEP panel is important and will contribute to advancing development studies.

## 2 Background

The survey concept originated in the research group FOR 756 'Impact of shocks on the vulnerability to poverty: consequences for development of emerging Southeast Asian economies', funded by the German Research Foundation (DFG) from 2007 to 2013. It involved several universities from Germany (Hannover, Göttingen, Gießen and Frankfurt). Since 2017, TVSEP is funded by the DFG as a long-term project titled 'Poverty dynamics and sustainable development: A long-term panel project in Thailand and Vietnam’. It is hosted by the Leibniz University Hannover and managed in cooperation with the University of Göttingen and since 2024 with the University of Cologne.

The purpose of this paper is to stimulate further use of data by providing information about the content of the surveys, the sampling and access to the data and by pinpointing some developments in rural Thailand and Vietnam since 2007. It is meant to be a guide and reference for scientific users. Apart from research, the data will ease policymaking with the goal of improving quality of life in the rural areas. Achieving high data quality is one of the major guiding principles of the data collection process.

## 3 Data Description

**Fig. 1:**
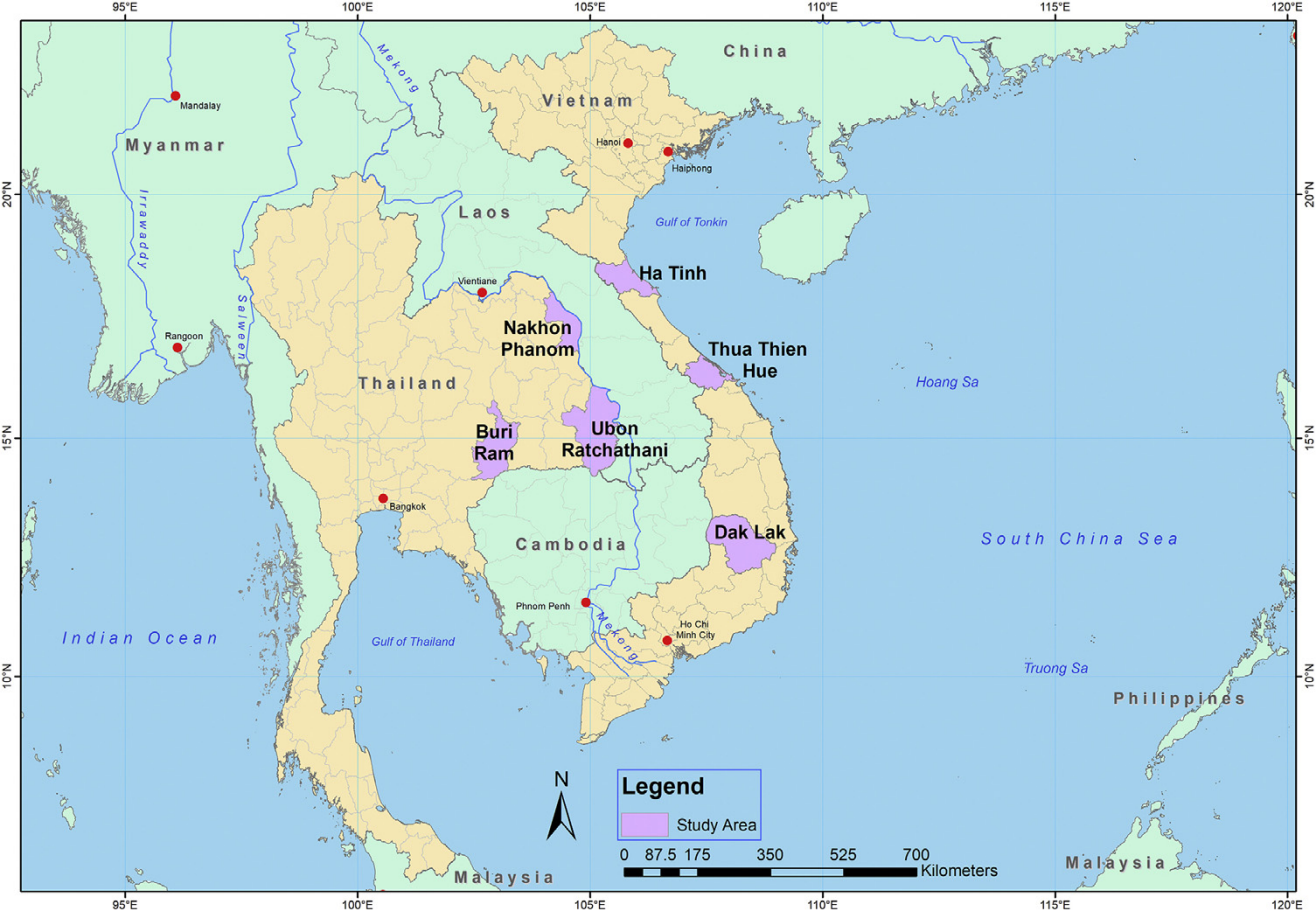
Study areas in Southeast Asia. Source: TVSEP.

Socioeconomic data from some 22,000 individuals in 4400 households in 440 villages were collected over time in three provinces of Thailand (Nakhon Phanom, Ubon Ratchathani, Buriram) and three provinces of Vietnam (Ha Tinh, Thua Thien Hue, Dak Lak) (Fig. 1).

**Fig. 2:**
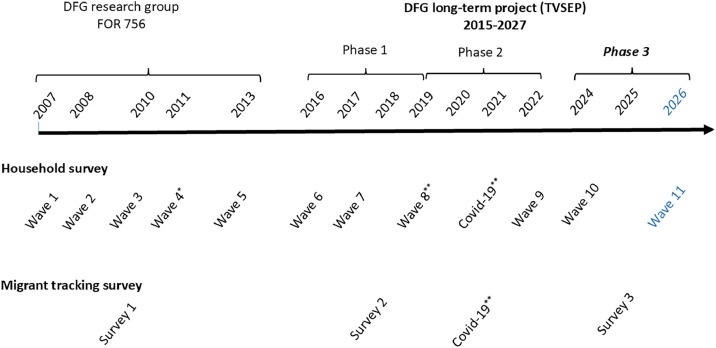
Timeline of the TVSEP project.

Source: Own compilation. *Note:* *1 province per country; **only Thailand; in blue: *planned survey*

The first TVSEP household survey took place in 2007, followed by further surveys of the same households in 2008, 2010, 2011 (in one province per country), 2013, 2016, 2017, 2019 (only in Thailand), 2022 (in Vietnam only in 2 provinces) and in 2024. A further household survey is planned for 2026 (Fig. 2).

The central survey instrument is the household questionnaire, with responses from the household head. However, TVSEP collects data not only on the households but also on the individuals of the households which expands the research potential.

The household questionnaire includes ten sections and provides around 800 questions.^1^ In sections 0 and 1, general information on the households is collected such as the location (i.e., province, district, sub-district/commune) where the households live. Furthermore, households are informed about the project, their anonymity and the confidential treatment of their data, and they are requested to give their consent to participate in the interviews. Section 2 collects information on the household members, their age, education, health, and occupations. In section 3, shocks and risks, as well as questions on well-being and aspirations, are included. Section 4 collects data on income generated from land, crops, livestock, fishing, hunting, and collecting natural resources, while sections 5 and 6 refer to wage employment and non-farm self-employment. Section 7 focuses on borrowing, lending, public transfers, and other payments received from the household as well as insurances. Section 8 refers to household expenditures and consumption, while section 9 collects information on assets and household wealth, including housing conditions, access to ICT, but also locus of control and personal satisfaction. Section 10 focuses on character traits.

In several years, the household surveys have been complemented by interviews with village heads. The survey instrument consists of six sections. The first section asks for some general survey information such as location or position of respondent. Section 2 refers to information such as number of villagers, foreigners or seasonal workers living in the village, their age structure and social problems in the village such as unemployment, gambling and drinking or illegal activities. Section 3 collects information about the infrastructure and institutions such as distance to cities or markets, access to public services, social and cultural activities or management of community resources. Section 4 refers to employment questions such as number of enterprises in the village, or the main occupations of households. Section 5 focuses on agriculture: the major agricultural activities, the availability of irrigation or prices of certain inputs. The last section 6 collects information on economic and environmental conditions, such as the number of poor versus rich people in the community, or the type and number of shocks in the last 12 months i.e. due to droughts, floods, pollution or erosion and government transfers to the village. Village head surveys have been conducted in 2007, 2010, 2011 (in one province per country), 2013, 2016, 2022 (only in Thailand) and in 2024. Another village head survey is planned for 2026.

In addition, migrant tracking surveys took place in both countries. These collected information from the migrants of the rural households and were conducted in the years 2010, 2018, 2021 (only in Thailand) and 2025. In 2021, the migrant tracking survey complemented a special COVID-19 household survey and was conducted by phone. All other migrant tracking surveys were conducted by face-to-face interviews. Their survey instrument consists of 12 sections and has a similar structure as the household questionnaire. It focuses on migration history, job history, living conditions of the migrants, shocks and risks, remittances, expenditures, and wealth. It also includes questions on personality traits and ICT knowledge and use. Migrants in Thailand are mainly interviewed in the Greater Bangkok Area, while in Vietnam, it was Ho Chi Minh City (HCMC) and surroundings as well as Da Nang City and in 2025 also in Hanoi. Migrants are interviewed by trained interviewers at their home or work place.

The following subsections outline some microeconomic trends and empirical research output on the basis of the TVSEP panel data. We focus on the last two decades which corresponds more or less with the length of the TVSEP panel survey and look more in detail into (i) age, gender and education of survey households; (ii) growth, income and poverty; and (iii) crises and shocks.

**Fig. 3:**
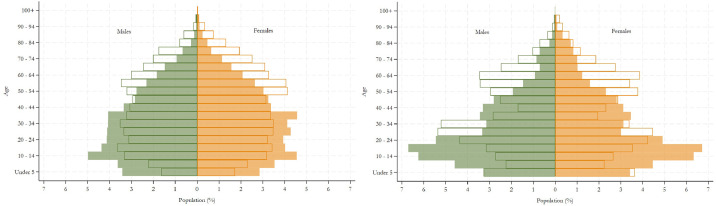
Population dynamics: Thailand (left) and Vietnam (right) (2007, 2024).

*Note:* Transparent overlay bars represent Thai and Vietnamese rural populations in 2024; All reported members in the reference period; Sample weight is applied.

The population pyramids (Fig. 3) show the age and gender distribution of the rural TVSEP sample population for Thailand and Vietnam for 2007 in comparison with 2024, with the latter being represented by the transparent overlay bars. Thailand’s population pyramid has a narrow base with fewer very young people and large shares of increasingly older age groups, reflecting that of developed countries with low birth rates and aging populations. In comparison, the proportion of young people in Vietnam is relatively higher than in Thailand, while the proportion of older people is comparatively lower. This reflects the economic potential and dynamism of the working population, as a large young population creates economic opportunities, the working-age population increases productivity, and the older population is manageable. Thus, the age structure reflects a more rapid economic growth in Vietnam, compared to Thailand.

Fig. 3 also shows declining shares of young people and rising shares of older age groups for Vietnam between 2007 and 2024. At the same time, it can be observed that the age groups between 35 and 50 have significantly declined in the survey regions over time, indicating rural-urban migration of the working-age people, leaving behind the elderly and the very young. This migration trend among working-age household members is much more pronounced in Vietnam than in Thailand.

**Fig. 4:**
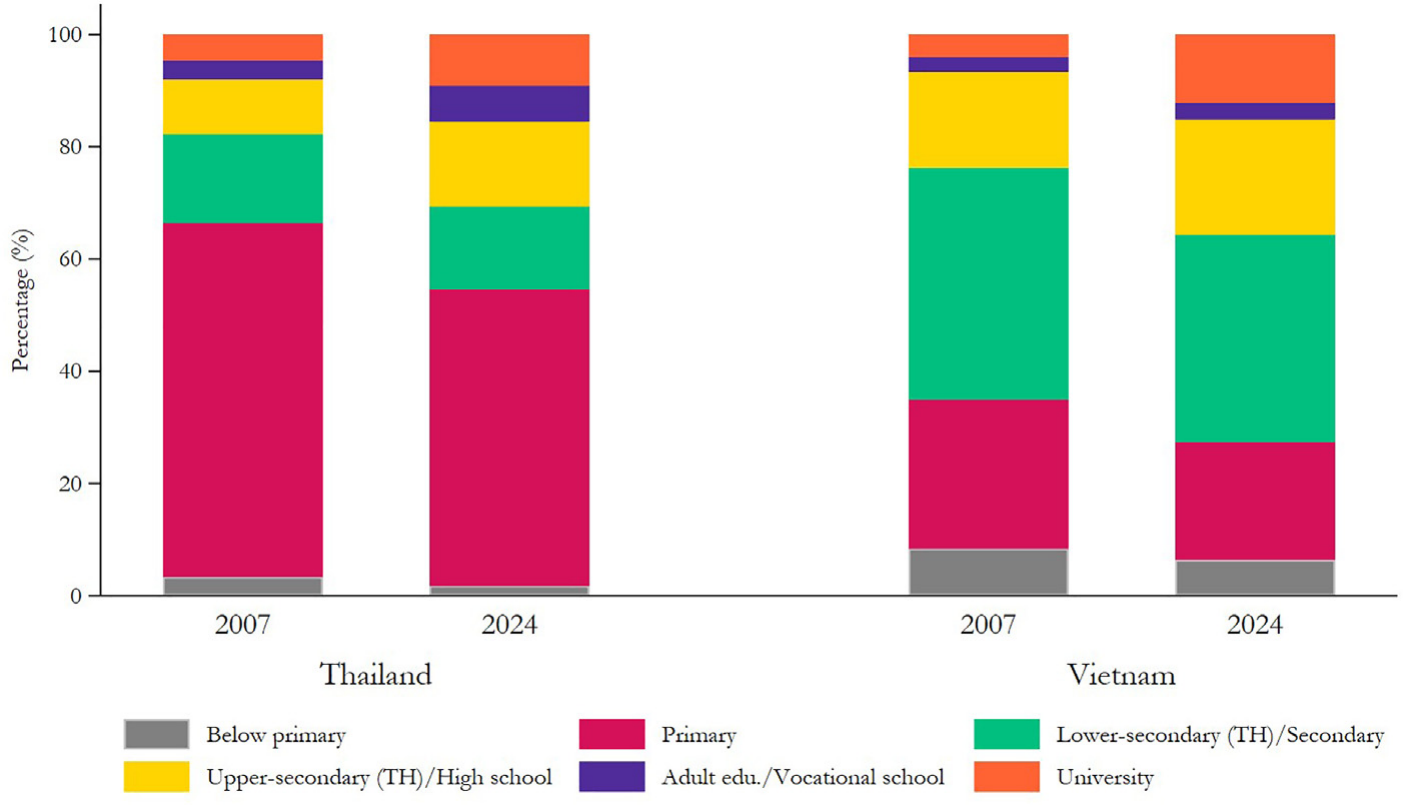
Change of educational levels in Thailand and Vietnam, 2007 and 2024.

*Note:* Sample weight is applied.

With respect to education, in Thailand, the share of people without any formal education was very low (around 3%) in 2007 and was even further reduced in 2024. In Vietnam, this share is significantly higher, amounting to around 12% in 2007 and falling to 8% in 2024 (Fig. 4). The share of people with only primary education is significantly higher in Thailand than in Vietnam, while the share of people with lower- and upper-secondary education is significantly higher in Vietnam than in Thailand. In both countries, the share of people with university degrees increased over time, but much more significantly in Vietnam than in Thailand. Higher education levels of households in the rural provinces have resulted in tangible outcomes for income generation and poverty reduction, as will be shown in the following subsection.

**Fig. 5:**
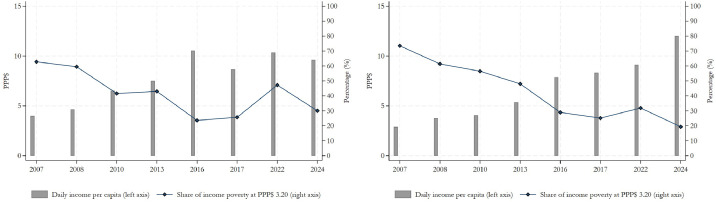
Income and poverty trends in Thailand (left) and Vietnam (right), 2007–2024 (threshold of PPP$ 3.20).

*Note:* In Vietnam: there were only Dak Lak and Ha Tinh in 2022; Sample weight is applied.

With respect to growth, income and poverty, TVSEP data show that income poverty declined significantly and daily per capita income increased between 2007 and 2024 in the respective rural regions, but this trend was less continuous in Thailand than in Vietnam and was interrupted in both countries by the COVID-19 pandemic (Fig. 5). More specifically, in 2007, around 65% of the surveyed households in Thailand were living below the income poverty line of PPP$ 3.20. This rate fell to around 25% in 2016, then rose sharply to almost 50% in 2022 due to the COVID-19 pandemic, before falling again to 30% in 2024. In Vietnam, around 75% of our surveyed households were living below the poverty line of PPP$ 3.20 in 2007. Within a decade, the rate dropped to around 25% (2017), increased slightly to around 32% in 2022 due to the COVID-19 pandemic, and fell again, reaching a value of around 20% in 2024. It can be concluded that while the overall welfare situation improved in the rural study areas, poverty persists in both countries.

**Fig. 6:**
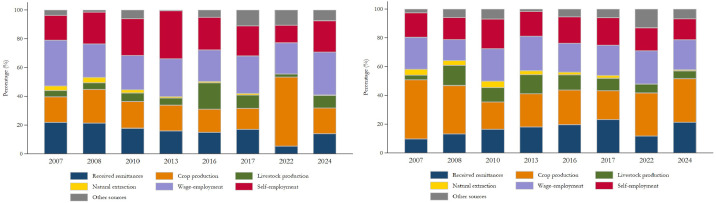
Development of income shares in Thailand (left) and Vietnam (right), 2007–2024.

*Note:* In Vietnam: there were only Dak Lak and Ha Tinh in 2022; Sample weight is applied.

Fig. 6 shows the development of income shares from different sources of rural households over time. The main sources include income from crop production, wage employment and self-employment. Additional sources cover remittances, income from livestock production and natural resources, as well as other sources. Major differences between the two countries exist with respect to the income from crop production (in orange). In Thailand, a slight drop occurred from 2008 to 2017. During the COVID-19 pandemic, the importance of farming as a major income source massively increased, but fell again thereafter. In Vietnam, there was a decline in income from crop production from 2007 to 2010, followed by slight fluctuations in the subsequent years. During the COVID-19 pandemic in 2022, agriculture gained relative importance and remained at this level in 2024. Wage employment remained relatively stable in both countries, while self-employment experienced fluctuations, particularly in Thailand. The relative importance of remittances (in blue) decreased for almost a decade from 2007 to 2016 in Thailand, and fluctuated since then. In Vietnam, there was a steady increase of remittances from 2007 to 2024, with an interruption during the COVID-19 pandemic. The drop in remittances during the COVID-19 pandemic was much more significant in Thailand than in Vietnam.

**Fig. 7:**
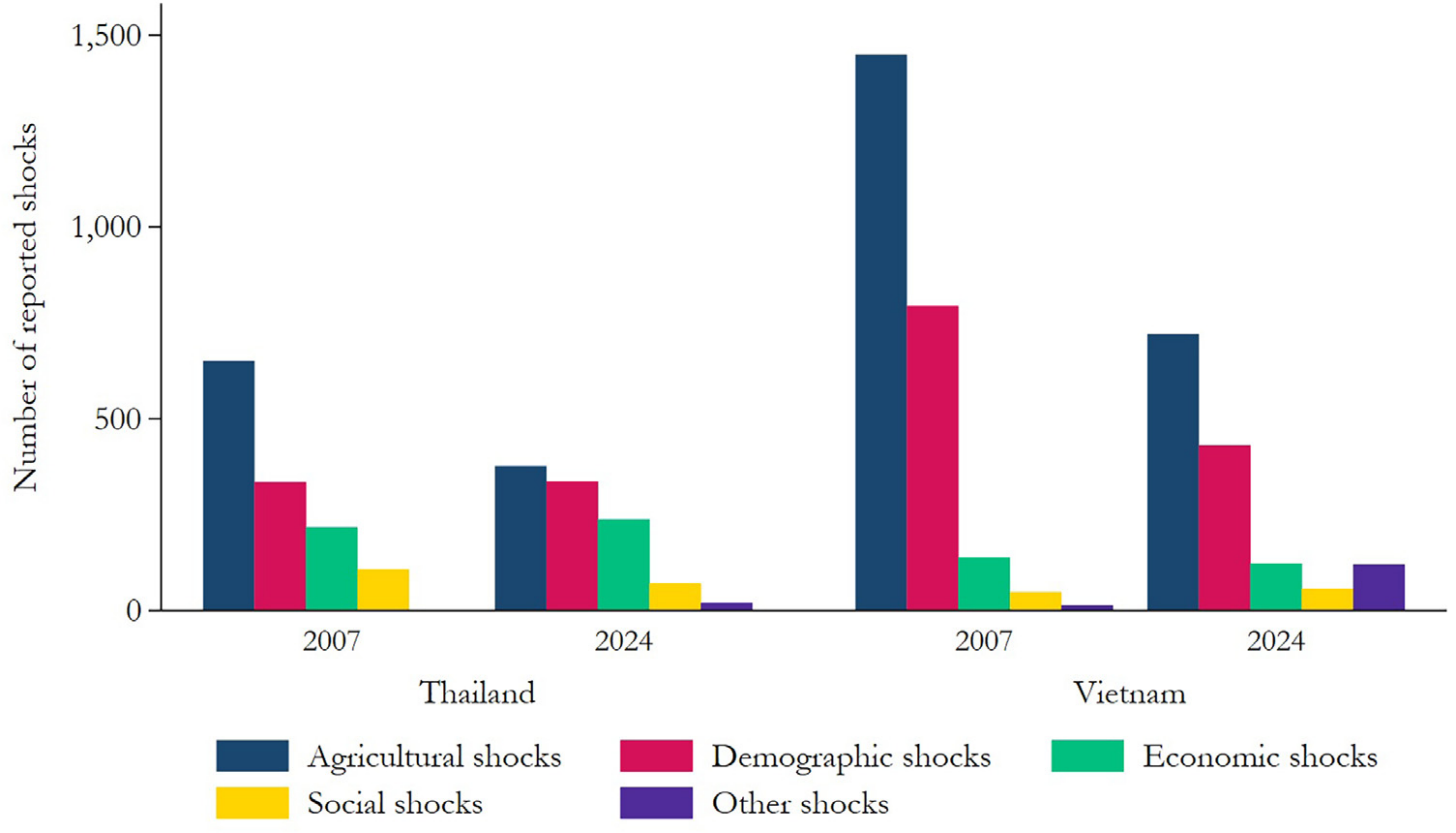
Situation of reported shocks in Thailand (left) and Vietnam (right) in 2007 and 2024.

*Note:* Number of reported shocks in the past 24 months of the survey years, between 2005 and 2007 or between 2022 and 2024.

With respect to crises and shocks in Thailand and Vietnam, data on self-reported shocks experienced over the last 12 months by the rural households were collected. These shocks encompass (i) agricultural shocks such as droughts, floods or storms, crop and livestock pests and diseases, but also (ii) demographic shocks such illness or death of household member or household member has left home, (iii) economic shocks including business failure, job loss, increasing input or decreasing output prices, (iv) social shocks such as conflicts with neighbors, and (v) other shocks such as crime. Fig. 7 compares the number of different reported shocks in Thailand and Vietnam in 2007 and 2024. Agricultural and demographic shocks proved to be most relevant in both countries, but more so in Vietnam than in Thailand. Economic and social shocks were slightly more often reported by households in Thailand than in Vietnam. In Thailand, the reported number of agricultural and also social shocks was higher in 2007 than in 2024, whereas the demographic and economic shocks increased a little. In Vietnam, agricultural and demographic shocks were much more often reported in 2024 than in 2007, whereas the social and other types of shocks increased a little. However, the comparison between the two years 2007 and 2024 does not allow yet any conclusions to be drawn about trends.

## 4 Experimental Design, Materials and Methods

In Thailand, the provinces were purposively selected based on the following criteria: (i) low average per capita income, (ii) high dependence on agriculture, (iii) remoteness and peripheral location along the country’s borders (either with Laos or Cambodia), (iv) underdeveloped infrastructure and (v) risky conditions for crop production due to floods, storms, or droughts. To identify subdistricts/communes, villages and eventually households for our panel interviews, a 3-stage cluster sampling procedure was applied. In the first stage, subdistricts were stratified in peri‑urban and rural subdistricts. To make sure that the rural population is covered proportionally, a systematic random sample was taken based on a list ranked by population density. In the second stage, two villages per subdistrict were selected from each of the sample subdistricts with a probability proportional to population size. In the third stage, a fixed-size sample of ten households was systematically selected from a list of households sorted by household size with equal selection probability. More details on the sampling and the formula can be found in [1].

In contrast to the sample from Thailand, the three Vietnamese provinces were selected based on agro-ecological zones (coastline, lowlands, and highlands). The most central province is Thua Thien Hue, which was the most developed of the three provinces in 2007 when the panel started. The northern province of Ha Tinh borders Laos via a long mountain range and was the least developed of the three TVSEP provinces. The third province, Dak Lak, is landlocked and located in the central highlands. It is characterized by a high proportion of ethnic minorities and is known for its coffee plantations, which date back to the French colonial era. Then, for the first stage, the population share of each district was used as a weighting factor for selecting communes (corresponding to subdistricts in Thailand), as no list of population densities was available. In the second stage, villages were selected with a probability proportional to their population size. The third stage was again a systematic random sample, which was drawn with equal probability from the village household lists sorted by household size [1].

The sampling procedure results in a sample of 2200 households in each country. Of these 2200 households, 2186 (99.4%) households were surveyed in the first wave in Thailand and 2195 (99,8%) households were surveyed in Vietnam in 2007 (Table 1). As expected, given the longitudinal nature of the panels, the response rate declined over the course of the panel. Accordingly, by 2017, the response rates dropped to 87% in Thailand and 86% in Vietnam. With these numbers, the cumulative attrition rate of the panel in both countries exceeded 10% of households, so that a replenishment of sample households was carried out in 2019 in Thailand with 2199 interviewed households and 2024 in Vietnam with 2198 interviewed households. In Thailand, the response rate during the first survey after the sample replenishment in 2020 amounted to 97,4%, in 2022 to 95,6% and in 2024 to 95,2%. Table 1 provides details on the numbers of sampled and successfully interviewed households and the attrition rates over all panel waves. Attrition derives e.g. from households moving to other places, household members dying, or survey fatigue. Overall, the attrition rates in the TVSEP project are quite low, which is impressive given that (i) the panel has been ongoing for a long time and (ii) the difficulties of collecting data from rural populations in developing countries.

**Table 1:** Number of households in each survey wave and attrition rates, 2007–2024.

		Original sample	Wave 1 - 2007	Wave 2 - 2008	Wave 3 – 2010	Wave 4 – 2011^b^	Wave 5 – 2013^c^	Wave 6 - 2016	Wave 7 - 2017	Wave 8 – 2019^a^	Covid-Survey 2020	Wave 9 - 2022	Wave 10 - 2024^a^
Thailand	Buriram	820	819	799	792	N/A	761	734	720	820	810	789	774
	Ubon Ratchathani	980	970	950	934	916	874	863	865	979	945	934	929
	Nakhon Phanom	400	397	387	379	N/A	361	344	329	400	386	378	391
	**Whole Thailand**	**2200**	**2186**	**2136**	**2105**	**916**	**1996**	**1941**	**1914**	**2199**	**2141**	**2101**	**2094**
Vietnam	Ha Tinh	720	720	713	701	N/A	659	634	641	N/A	N/A	604	720
	Thua Thien Hue	720	718	699	683	672	648	614	611	N/A	N/A	N/A	720
	Dak Lak	760	757	736	715	N/A	703	644	646	N/A	N/A	623	758
	**Whole Vietnam**	**2200**	**2195**	**2148**	**2099**	**672**	**2010**	**1892**	**1898**	N/A	N/A	**1227**	**2198**
	**Whole sample**	**4400**	**4381**	**4284**	**4204**	**1588**	**4006**	**3833**	**3812**	**2199**		**3328**	**4292**
Attrition rate compared with the original sample													
	Thailand	-	0.6%	2.9%	4.3%		9.3%	11.8%	13.0%	-	2.6%	4.5%	4.8%
	Vietnam	-	0.2%	2.4%	4.6%		8.6%	14.0%	13.7%	-			
	**Whole sample**	**-**	0.4%	2.6%	4.5%		9.0%	12.9%	13.4%	**-**			
Attrition rate compared with the previous wave													
	Thailand		-	2.3%	1.5%		5.2%	2.8%	1.4%		2.6%	1.9%	0.3%
	Vietnam		-	2.1%	2.3%		4.2%	5.9%	−0.3%				
	**Whole sample**		**-**	2.2%	1.9%		4.7%	4.3%	0.5%				

*Note:*.

a The replenishment of households was conducted in 2019 in Thailand and 2024 in Vietnam.

b only one province in each country.

## Limitations

The fourth wave of the household survey, conducted in 2011, covered only one province per country. In 2022, the household survey was only implemented in Thailand.

In 2021, the migrant tracking survey complemented a special COVID-19 household survey and was conducted by phone and only in Thailand. All other migrant tracking surveys were conducted by face-to-face interviews.

## Ethics Statement

This study was approved by the Partner Universities in Thailand and Vietnam (approved number: EEPSEA-HTH-300,925-01). Informed consent was obtained from respondents at the beginning of the interviews.

## CRediT Author Statement

**Ulrike Grote:** Writing – original draft, Writing – review & editing, Visualization, Project administration. **Trung Thanh Nguyen:** Writing – review & editing, Data curation, Project administration. **Manh Hung Do:** Writing – review & editing, Data curation, Visualization.

## Data Availability

Leibniz University HannoverTVSEP (Original data).

## Appendix Supplementary materials

Image, application 1

## Footnotes

1 https://www.tvsep.de/en/.
