# Supplementary figures and images for: TVSEP: A comprehensive long-term panel dataset on shocks, welfare and agriculture in rural Thailand and Vietnam
Source: Data Brief. 2026 Jun 19;67:112993. doi: 10.1016/j.dib.2026.112993 (PMC13343005; doi:10.1016/j.dib.2026.112993)

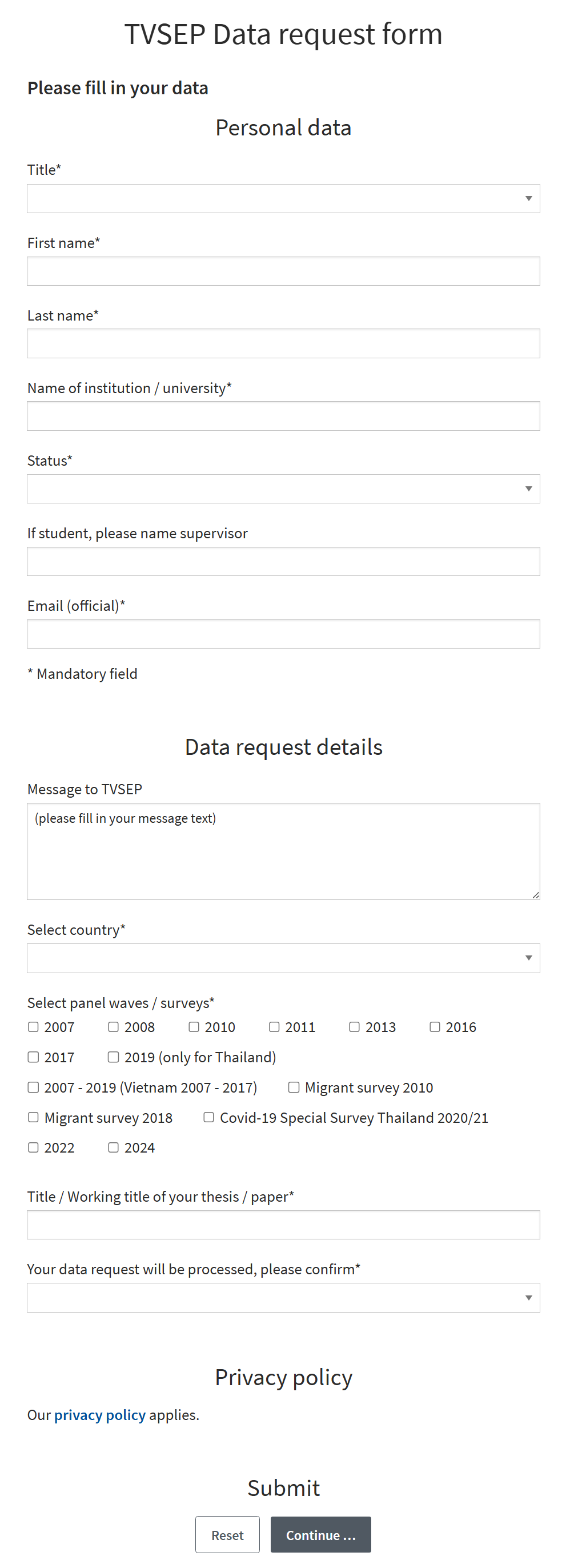

Supplement: Supplementary file 1 [file mmc1.docx]
